# Molecular investigation of bacterial communities: Data from two frequently used surfaces in the São Paulo Institute of Tropical Medicine

**DOI:** 10.1016/j.dib.2016.05.064

**Published:** 2016-06-02

**Authors:** Tairacan Augusto Pereira da Fonseca, Rodrigo Pessôa, Sabri Saeed Sanabani

**Affiliations:** a Clinical Laboratory, Department of Pathology, LIM 03, Hospital das Clínicas, School of Medicine, University of São Paulo, São Paulo 05403 000, Brazil; b São Paulo Institute of Tropical Medicine, University of São Paulo, São Paulo 05403 000, Brazil

## Abstract

This article contains data on the bacterial population of two frequently used surfaces in the São Paulo Institute of Tropical Medicine (ITM) using the Illumina sequencing for massive parallel investigation of the bacterial 16S ribosomal RNA gene. Surface samples were obtained from restroom surfaces and the fingerprint door clock system. Mothur package and Shannon-ace-table.pl software programs (Chunlab Inc.: Seoul, Korea) were used to compute the diversity indices of bacterial community. The sequencing data from both surfaces have been uploaded to Zenodo: http://dx.doi.org/10.5281/zenodo.47709

## **Specifications Table**

**Table:** 

Subject area	*Biology*
More specific subject area	*Microbiome*
Type of data	*Text files: sequences*
How data was acquired	*Culture-independent Illumina massively parallel sequencing approach of the 16S rRNA genes using the Illumina sequencing-by-synthesis method (MiSeq platform).*
Data format	*Sequencing*
Experimental factors	*Bacterial genomic DNA was extracted and used as a template to amplify the V4 region of the 16S rRNA gene. The amplicons were molecularly barcoded, pooled, and sequenced by a paired-end protocol (Illumina).*
Experimental features	*Illumina massively parallel sequencing of the 16S rRNA gene libraries and subsequent analysis.*
Data source location	*São Paulo, Brazil*
Data accessibility	*Data is within this article and have been uploaded to Zenodo* (http://dx.doi.org/10.5281/zenodo.47709).

## Value of the Data

• Exploration of bacterial communities on these surfaces in our institute and other establishments will advance our knowledge of microbial trafficking and colonization in these systems.
• These data are publically available and their access permits researchers to conduct further analyses based on their own studies with newly developed bioinformatics packages.
• The data presented here underscores the need to increase public awareness on the importance of personal hygiene and proper hygienic practices of inanimate surfaces to reduce the transmission of disease-causing organisms.

## 1 Data

All sequence data described here are available in the online Zenodo repository: http://dx.doi.org/10.5281/zenodo.47709. The two sets of massively parallel sequence (MPS) data represent the bacterial communities from the restroom surfaces and the fingerprint door clock system (FDLS) in the São Paulo Institute of Tropical Medicine (ITM).

## 2 Experimental design, materials, and methods

Surfaces of four IMT-FDLS that are frequently used by the institute׳s professionals were swabbed and included in this study. Samples were also obtained from 12 surfaces in two male and two female restrooms including door handles into and out of the restroom, toilet seats, faucet handles, and hands-toilet flush handles. All surfaces were sampled and treated as previously described [1].

Genomic DNA was extracted from each swab using the PowerSoil DNA kit (MO BIO Laboratories™: Carlsbad, CA, USA) as per the manufacturer׳s instructions. After the extraction, DNA from each surface group was pooled together, and the pooled DNA was submitted to the amplification of the V4 region of the 16S rRNA gene using the previously published primers Bakt_341F/Bakt_805R [2] according to the conditions previously described [1], [3]. Purification of the amplified products were performed using the Freeze N Squeeze DNA Gel Extraction Spin Columns (Bio-Rad: Hercules, CA, USA), following the recommendations of the manufacturer. After purification and quantification on a Qubit 2.0 Fluorometer (Life Technologies: Carlsbad, CA, USA), the amplicons from each surface group were pooled at equimolar concentration, and diluted to 4 nM. Indexing of DNA and preparation of libraries were performed as previously reported (our references). The prepared library was finally loaded on an Illumina MiSeq cartridge for paired end 300 sequencing.

Image analysis, base calling, and data quality assessment were initially performed on the MiSeq instrument (San Diego, CA, USA). Sequence cleaning and analysis was conducted according to the recently published methods by our group [1], [3]. Briefly, the PANDAseq v.2.9 software was used to pair the MiSeq forward and reverse reads [4] with default parameters. The UCHIME algorithm [5] was used to detect and remove any potential recombinant sequences from the generated data. The EzTaxon-e database [6] adjusted at a 97% cutoff of sequence similarity identity was used to define the taxonomic classification of each read. The Chao1 estimation and the Shannon diversity index at a 3% distance were used calculate the richness and diversity of samples. The Mothur and Shannon-ace-table.pl software programs (Chunlab Inc.: Seoul, Korea) were used to compute the diversity indices of bacterial community. The Fast UniFrac [7] was used to estimate the overall phylogenetic distance between communities. This distance was visualized using principal coordinate analysis (PCoA). Shared OTUs operational taxonomic units (OTUs) were identified using XOR analysis of CL community program v3.43 (Chunlab Inc.: Seoul, Korea).

**Fig. 1:**
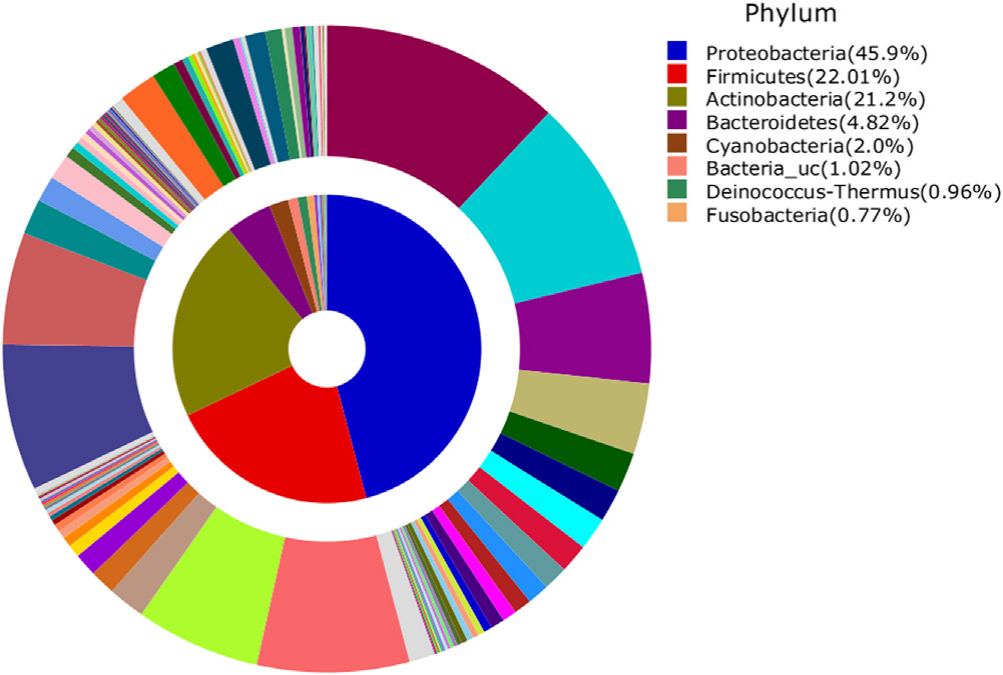
Average composition of bacteria from all samples (inner area: Phylum, outer area: Family). Phyla and Families with more than 1% of their proportion were represented.

**Fig. 2:**
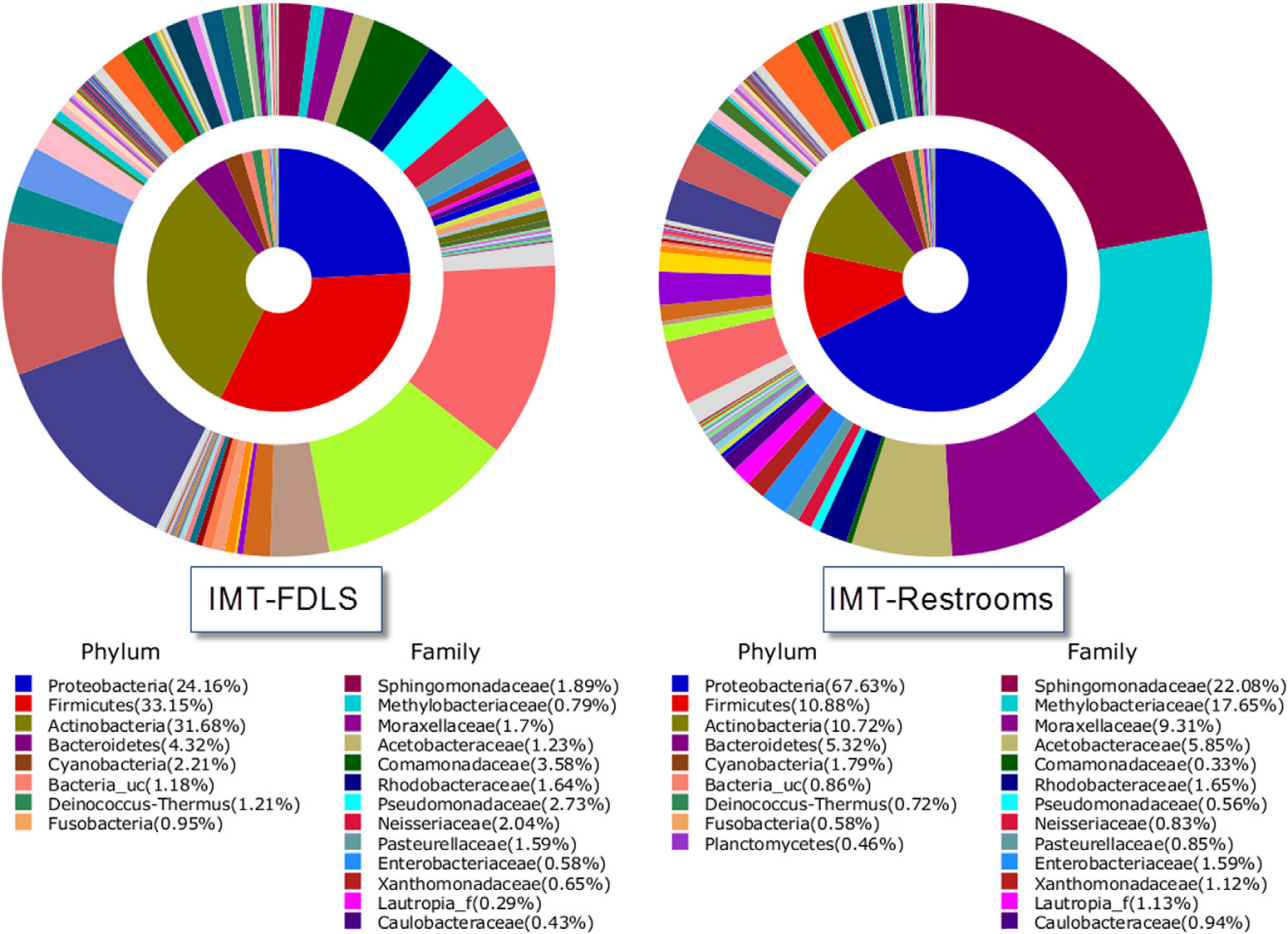
Average composition of bacteria from each sample (inner area: Phylum, outer area: Family). Only bacterial phyla and families that had a relative abundance of 1% or greater are presented.

The most abundant OTUs at phylum and family levels that accounted for more than 1% of all sequences, IMT-FDLS and, IMT-restrooms are shown in Fig. 1, Fig. 2. The six OTUs of the most abundance species associated with the two sample libraries are provided in Table 1.

## Figures and Tables

**Table 1:** Identities of the six most abundant OTUs in the bacterial communities.

**Abundance order**	**Taxa (% of abundance)**	
	**IMT-FDLS**	**IMT-restrooms**
1	*Propionibacterium acnes* (7.9%)	*Moraxella osloensis* (5.6%)
2	*Streptococcus dentisani* (3.5%)	*Methylobacterium phyllostachyos* (5.4%)
3	*Staphylococcus epidermidis* (3%)	EU440723_s (4.2%)
4	*Staphylococcus capitis* (2.4%)	*Sphingomonas ginsenosidimutans* (3.8%)
5	Streptococcus_uc m(1.9%)	*Roseomonas mucosa* (3.7%)
6	Streptococcaceae_uc_s (1.9%)	Sphingomonadaceae_uc_s (3.4%)
